# The iPREFACE score is useful for predicting fetal acidemia: A retrospective cohort study of 113 patients who underwent emergency cesarean section for non-reassuring fetal status during labor

**DOI:** 10.1016/j.xagr.2024.100343

**Published:** 2024-04-04

**Authors:** Ayumu Ito, Eijiro Hayata, Hikari Kotaki, Makiko Shimabukuro, Mayumi Takano, Sumito Nagasaki, Masahiko Nakata

**Affiliations:** 1 Department of Obstetrics and Gynecology, Faculty of Medicine, Toho University, Tokyo, Japan (Drs Ito, Hayata, Kotaki, Shimabukuro, Takano, Nagasaki, and Nakata); 2 Department of Obstetrics and Gynecology, Toho University Omori Medical Center, Tokyo, Japan (Drs Ito, Hayata, Kotaki, Shimabukuro, Takano, Nagasaki, and Nakata).

**Keywords:** acidemia, acidosis, cardiotocography, cesarean delivery, deceleration, fetal heart rate monitoring, neonatal asphyxia, nonreassuring fetal status, rapid delivery, umbilical cord artery blood

## Abstract

**BACKGROUND:**

The iPREFACE score may aid in predicting fetal acidemia and neonatal asphyxia in emergency cesarean and vaginal deliveries, which may improve labor management precision in the future.

**OBJECTIVE:**

This study aimed to assess the score use of the iPREFACE as an objective indicator of the need for rapid delivery in cases of repeated abnormal waveforms without concurrent indications for immediate medical intervention during labor.

**STUDY DESIGN:**

This retrospective cohort study was conducted among term (37+ 0 days to 41+6 days) singleton pregnant women who underwent emergency cesarean delivery owing to a nonreassuring fetal status. The integrated score index to predict fetal acidemia by intrapartum fetal heart rate monitoring–decision of emergency cesarean delivery score, calculated from a 30-minute cardiotocography waveform before the decision to perform emergency cesarean delivery, and the integrated score index to predict fetal acidemia by intrapartum fetal heart rate monitoring–removal of cardiotocography transducer score, calculated from a 30-minute cardiotocography waveform before cardiotocography transducer removal, were employed. The primary outcome was the assessment of the predictive ability of these scores for fetal acidemia, whereas the secondary outcomes were differences in umbilical artery blood gas findings and postnatal outcomes between the 2 groups, divided by the cutoff values of the integrated score index to predict fetal acidemia by intrapartum fetal heart rate monitoring–removal of cardiotocography score.

**RESULTS:**

The integrated score index to predict fetal acidemia by intrapartum fetal heart rate monitoring–decision of emergency cesarean delivery and integrated score index to predict fetal acidemia by intrapartum fetal heart rate monitoring–removal of cardiotocography transducer scores demonstrated the capability to predict an umbilical artery blood pH of <7.2. The integrated score index to predict fetal acidemia by intrapartum fetal heart rate monitoring–decision of emergency cesarean delivery and −removal of cardiotocography transducer score, with cutoff values of 37 and 46 points, respectively, exhibited an area under the receiver operating characteristic curve of 0.82 and 0.87, respectively. The integrated score index to predict fetal acidemia by intrapartum fetal heart rate monitoring–removal of cardiotocography transducer group with ≥46 points had higher incidence rates of an umbilical cord artery blood pH of <7.2, <7.1, and <7.0 and neonatal intensive care unit admissions for neonatal asphyxia.

**CONCLUSION:**

The integrated score index to predict fetal acidemia by intrapartum fetal heart rate monitoring, derived from cardiotocography during an emergency cesarean delivery, may enable clinicians to predict fetal acidemia in cases of nonreassuring fetal status. Improved prediction of fetal acidemia and facilitation of timely intervention hold promise for enhancing the outcomes of mothers and newborns during childbirth. Prospective studies are warranted to establish precise cutoff values and to validate the clinical application of these scores.

**AJOG Global Reports at a Glance**

**Why was this study conducted?**

This study aimed to assess the use of the integrated score index to predict fetal acidemia by intrapartum fetal heart rate monitoring (iPREFACE score) as an objective indicator of the need for rapid delivery in cases of repeated abnormal waveforms during labor.

**Key findings**

The iPREFACE–decision of cesarean delivery (DCS) and iPREFACE–removal of cardiotocography transducer (RCT) scores demonstrated capability to predict umbilical artery blood pH of <7.2. An iPREFACE-RCT score ≥46 points group had higher incidence rates of umbilical cord artery blood pH <7.2, <7.1, and <7.0, and neonatal intensive care unit admissions for neonatal asphyxia.

**What does this add to what is known?**

The iPREFACE score, derived from cardiotocography during emergency cesarean delivery, may predict fetal acidemia in cases of nonreassuring fetal status, offering potential for improved outcomes through timely intervention. Prospective studies are needed to establish precise cutoff values and validate clinical application.

## Introduction

Cardiotocography (CTG), which was developed in 1958, is widely used worldwide to assess fetal well-being.^1^ However, after CTG was introduced to reduce the risk for fetal hypoxic encephalopathy, the number of births that lead to cerebral palsy has not decreased despite an increase in the cesarean delivery rate.^2^^,^^3^ Furthermore, the false-positive rate of CTG to predict cerebral palsy is as high as 99.8%,^4^ and a debate continues regarding the optimal method of delivery management based on CTG data.

The guidelines on delivery monitoring using CTG include a 3-tier classification of fetal heart rate waveforms^5^, ^6^, ^7^ and a 5-tier classification proposed by Parer and Ikeda.^8^ The latter classification was adopted in Japan.^9^ Based on the 5-tier classification by the Japanese Society of Obstetrics and Gynecology (JSOG),^9^ fetal heart rate waveforms are classified as follows: level 1, normal; level 2, subnormal; level 3, abnormal (ie, mild); level 4, abnormal (ie, moderate); and level 5, abnormal (ie, severe). The classification of fetal heart rate waveform levels defines the risk for fetal hypoxemia or acidemia by integrating fetal heart rate variability, baseline fetal heart rate, and decelerations. An increase in the level corresponds to an increase in risk. Levels 3 to 5 are defined as nonreassuring fetal status (NRFS) at delivery. In cases in which the fetal heart rate waveform is identified as level 5, interventions, such as vacuum delivery or emergency cesarean deliver (eCD), are recommended. Should a waveform categorized as level 3 or 4 persist, a skilled obstetrician should evaluate the pace and progress of delivery. If it is determined that continuation of vaginal delivery presents challenges, an eCD is advised. Delivery interventions are determined based on this classification. Several studies^10^, ^11^, ^12^, ^13^ have demonstrated that the 5-tier classification is more effective than the 3-tier classification for predicting fetal acidemia. Nevertheless, many CTG management methods, including the 5-tier classification, do not effectively identify fetal acidemia in cases in which the umbilical cord artery blood pH is <7.15.^14^ This factor highlights the limitations of the current CTG guidelines for predicting fetal acidemia.^14^ However, we previously reported an integrated score index to predict fetal acidemia by intrapartum fetal heart rate monitoring (iPREFACE score), which is derived from a 30-minute CTG waveform recorded before delivery.^15^^,^^16^ The iPREFACE score has demonstrated high predictive ability for fetal acidemia by quantifying fetal distress during delivery.

The 5-tier JSOG classification indicates that rapid delivery is recommended for level 5 waveforms.^9^ If a level 3 or 4 waveform is repeatedly observed, the delivery speed and progress should be periodically assessed. If vaginal delivery is deemed difficult, an eCD should be performed.^9^ However, no objective criteria to endorse timely intervention after the decision to perform an eCD exist for obstetricians to intervene when CTG shows abnormal level 3 or 4 waveform patterns. In many cases when CTG shows abnormal level 3 or 4 waveform patterns, the decision to perform an eCD often depends on the judgment of the attending obstetrician. Hence, an indicator that could be used to predict fetal acidemia and hypoxic encephalopathy, based on abnormal CTG waveform patterns, is needed. The aim of this study was to clarify the usefulness of the iPREFACE score as an objective indicator of predicting fetal acidemia and determining the need for rapid labor in cases of repeated abnormal level 3 or 4 CTG waveforms classified according to the 5-tier classification system.

## Materials and Methods

### Study design and patient population

This retrospective cohort study included women who given birth at the Toho University Omori Medical Center (Tokyo, Japan) from April 2017 to March 2021. The inclusion criteria were term (37+0 days to 41+6 days) singleton pregnant women who underwent eCD because of NRFS indicators during ongoing labor. This cohort included cases in which attempts at operative vaginal delivery were made. We excluded patients with cases of fetal growth retardation, fetal malformations, and fetal chromosomal abnormalities, and patients in situations in which the CTG waveform could not be continuously and accurately measured for 30 minutes before the time of the decision to perform an eCD.

The Toho University Omori Medical Center, situated in Tokyo, Japan, serves as a perinatal center, overseeing approximately 500 to 700 deliveries annually, with a CD rate of about 36% and an average interval from the decision for CD to delivery of around 69 minutes. For cases that required fetal delivery within 30 minutes or when neonatal resuscitation by the neonatology department was anticipated, CDs were performed in a specialized operating room adjacent to the delivery room on the 8th floor of Building 2. In other scenarios, the procedure was carried out in the central operating room on the 3rd floor of Building 3, accessed via a connecting corridor on the 2nd floor between Buildings 2 and 3, a distance of approximately 70 meters.

### Routine clinical intrapartum care using cardiotocography

**Figure 1:**
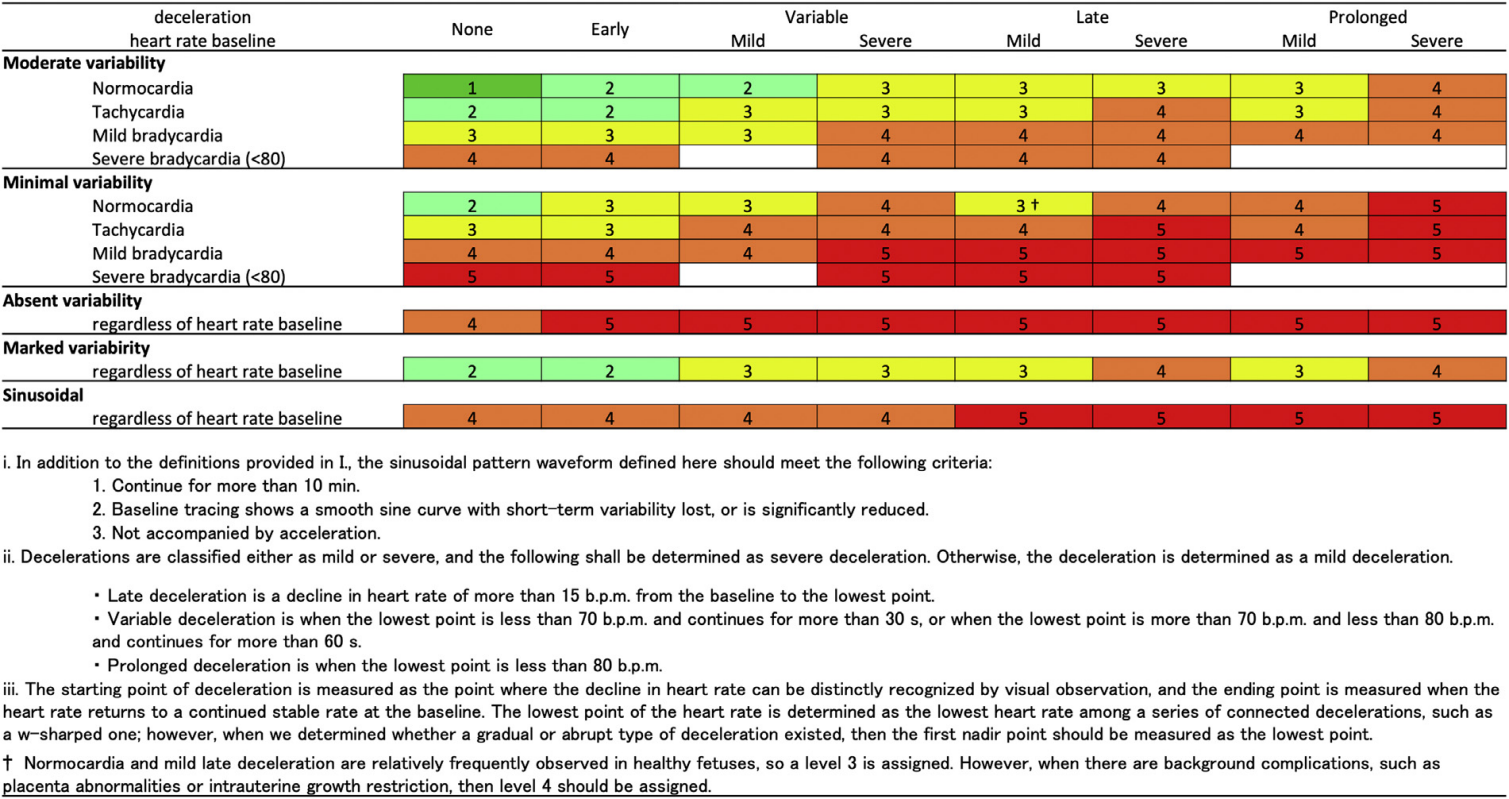
Risk levels of fetal heart rate waveforms in the five-tier classification. *b.p.m.*, beat per minute.

Management based on CTG during delivery was based on the 5-tier classification of fetal heart rate waveforms as defined by the JSOG (Figure 1).^9^^,^^15^ In the first stage of labor, when the fetal heart rate waveform was at level 1, the CTG transducers were placed for at least 20 minutes per session at intervals of ≤6 hours. When the patient was not wearing the CTG transducer, intermittent fetal heartbeats were checked at 15- to 90-minute intervals by using a handheld ultrasound Doppler device. When the fetal heart rate waveform was classified as a waveform other than level 1, continuous monitoring using CTG was performed unless a trained obstetrician, midwife, or nurse deemed the patient amendable to takeover and throughout follow-up.

Ito. Integrated score index to predict fetal acidemia by intrapartum fetal heart rate monitoring is useful for predicting fetal acidemia in emergency cesarean delivery for nonreassuring fetal status during labor. Am J Obstet Gynecol Glob Rep 2024.

### Diagnosis of nonreassuring fetal status and decision-making for expedited delivery

The diagnosis of NRFS during delivery and the decision for rapid delivery were based on the JSOG guidelines.^9^ NRFS was diagnosed when the fetal heart rate waveform was at levels 3 to 5. If the fetal heart rate waveform was diagnosed as NRFS at level 5, interventions such as vacuum delivery or eCD were performed. If a waveform at level 3 or 4 persisted, a trained obstetrician assessed the delivery pace and progress. If a physician deemed that to continue with vaginal delivery would be difficult, an eCD was performed. In these situations, the neonatologist monitored the initial response at birth.

### Calculating the iPREFACE score

**Figure 2:**
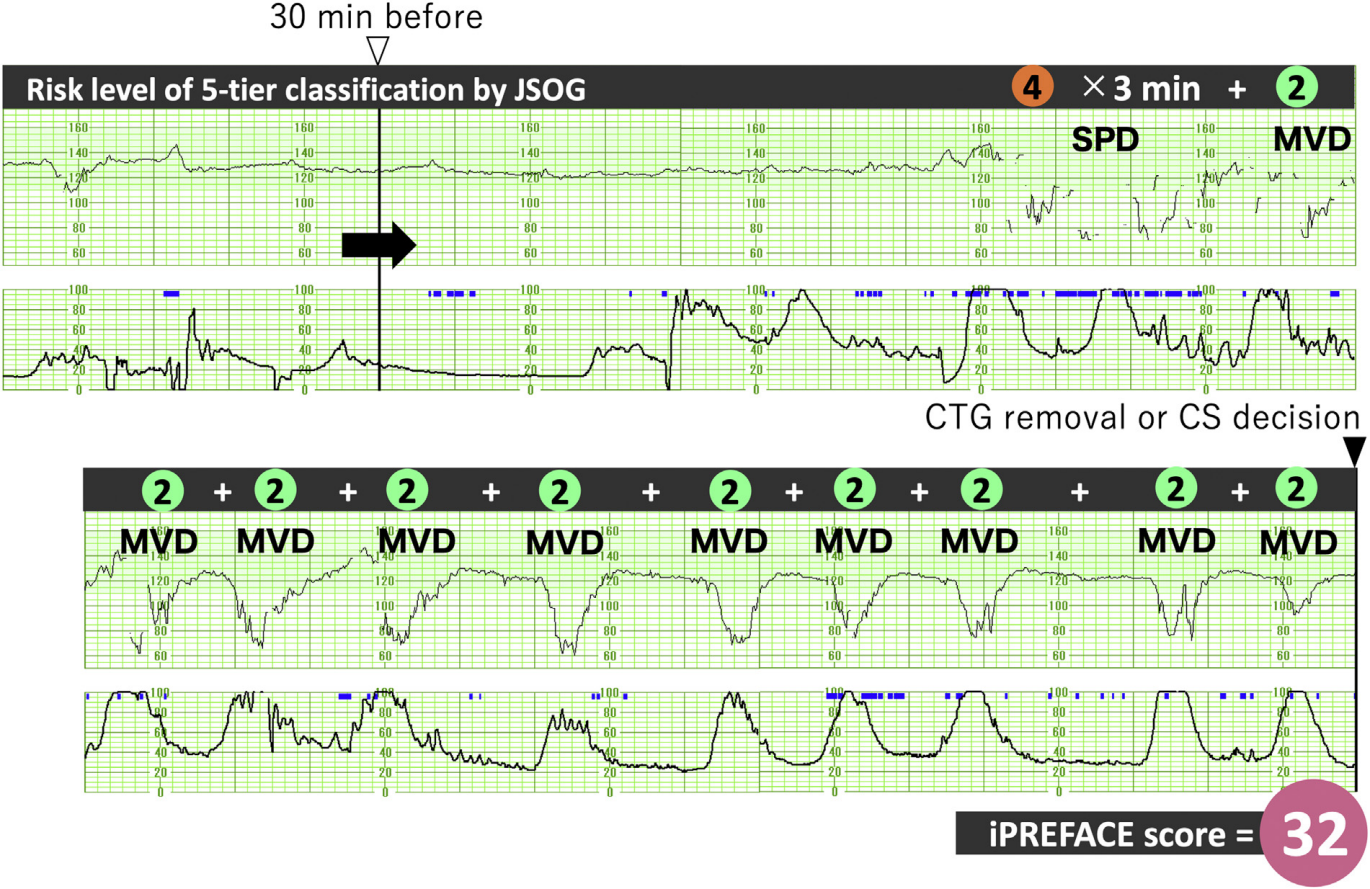
Example of the iPREFACE scoring process in this study *iPREFACE*, integrated score index to predict fetal acidemia by intrapartum fetal heart rate monitoring; *JSOG*, Japanese Society of Obstetrics and Gynecology; *MVD*, mild variable deceleration; *SPD*, severely prolonged deceleration.

The iPREFACE score was calculated by summating the fetal heart rate waveform levels for all detected decelerations,^9^ excluding prolonged decelerations, in the 30-minute CTG recording taken during labor.^15^^,^^16^ However, prolonged decelerations were determined by adding the products of the waveform levels and the duration (rounded to the nearest whole minute) of decelerations.^15^^,^^16^ Figure 2 illustrates how this scoring process was implemented.

Ito. Integrated score index to predict fetal acidemia by intrapartum fetal heart rate monitoring is useful for predicting fetal acidemia in emergency cesarean delivery for nonreassuring fetal status during labor. Am J Obstet Gynecol Glob Rep 2024.

**Figure 3:**
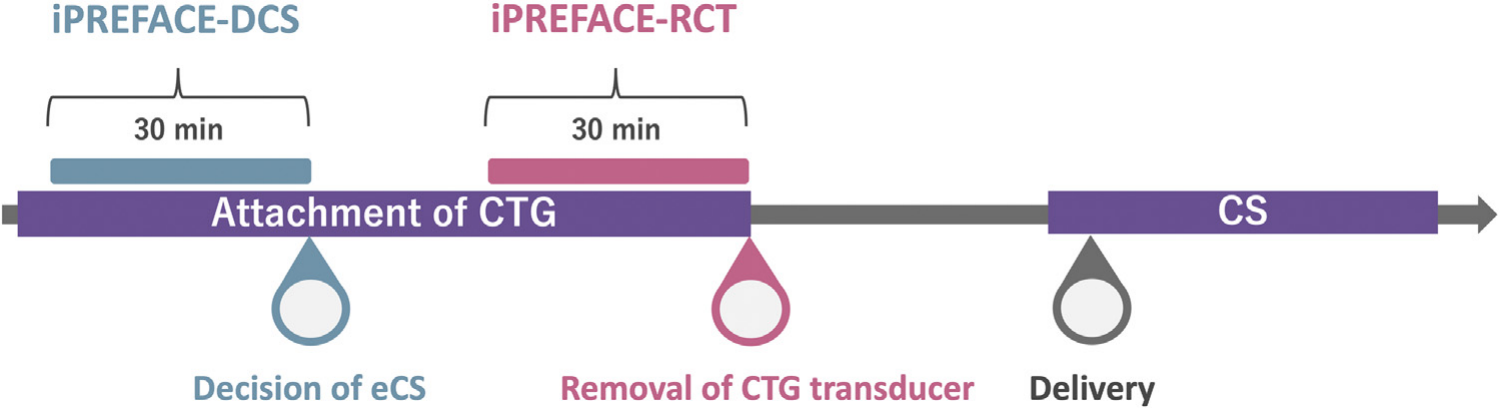
Definition of the iPREFACE score used in this study *CS*, cesarean delivery; *CTG*, cardiotocography; *DCS*, decision of cesarean delivery; *eCS*, emergency cesarean delivery; *iPREFACE*, integrated score index to predict fetal acidemia by intrapartum fetal heart rate monitoring; *RCT*, removal of CTG transducer.

In this study, we defined the iPREFACE score, which was retrospectively calculated using 30 minutes of CTG data before the time of the decision to perform an eCD as the iPREFACE–decision of emergency CD (DCS) score. We also retrospectively defined the iPREFACE score, calculated using 30 minutes of CTG data before the time of CTG monitor removal, as the iPREFACE–removal of CTG transducer (RCT) score (Figure 3). We did not evaluate interrater error in this study because previous studies have demonstrated that the intraclass correlation coefficient for 3 raters of the iPREFACE score was 0.91.

Ito. Integrated score index to predict fetal acidemia by intrapartum fetal heart rate monitoring is useful for predicting fetal acidemia in emergency cesarean delivery for nonreassuring fetal status during labor. Am J Obstet Gynecol Glob Rep 2024.

### Outcomes

First, we calculated the area under the curve (AUC) and cutoff values to assess the predictive ability of the iPREFACE-DCS and iPREFACE-RCT scores to detect fetal acidemia risk by using receiver operating characteristic (ROC) curves.

Second, in cases of fetal heart rate waveforms at levels 3 and 4, the iPREFACE-RCT scores were categorized into 2 groups, namely above the cutoff value and below the cutoff value. The patients’ characteristics and umbilical artery blood gas analysis results were subsequently compared between the 2 groups.

### Outcome variables and definitions

We defined fetal acidemia as an umbilical artery blood pH <7.2 based on clinical considerations. When using the iPREFACE score in a clinical setting, prioritizing neonatal safety at birth is essential. An umbilical cord artery blood pH <7.1 is associated with adverse neurologic events in 75% of newborns.^17^ Therefore, establishing an intervention threshold that ensures neonatal safety is crucial.

### Statistical analyses

All statistical analyses were conducted using SPSS, version 29 (IBM Corp., Armonk, NY). Between-group comparisons were conducted using the *t* test for parametric data and the Mann-Whitney *U* test for nonparametric data. The values among the 3 groups were compared using 1-way analysis of variance for parametric data and the Kruskal-Wallis test for nonparametric data. Proportions were compared using the chi-square and Fisher exact probability tests. We determined the cutoff values of the iPREFACE score to predict fetal acidemia in both groups by using the maximal Youden index abd selecting the point at which sensitivity + specificity – 1 was the maximum value on the ROC curves. We also calculated the sensitivity, specificity, and positive and negative predictive values. Statistical significance was set at a *P* value of <.05. In the design of this retrospective study, we calculated the required sample size based on the expected discriminative ability of the diagnostic test, as indicated by the AUC (0.80). To achieve a statistical power of 0.90 and to adhere to a significance level of 5% for 1-sided tests, while considering a disease prevalence ratio of 1:9 (disease present:disease absent), our calculations determined that a total sample size of approximately 80 participants was necessary. This estimation accounted for approximately 8 participants with fetal acidemia and approximately 72 participants without fetal acidemia. These sample size calculations were conducted using statistical power analysis with incorporation of the effect size derived from the specified AUC value.

### Ethics statement

This study was approved by the Ethics Committee of the Toho University Omori Medical Center (approval no. M22082) on July 4, 2021, and was conducted in accordance with the ethical standards outlined in the 1964 Declaration of Helsinki and its subsequent amendments or comparable ethical standards. Information regarding the study was published in an opt-out format on the hospital website, thereby allowing potential research participants the opportunity to refuse participation. The need for written informed consent was waived. In accordance with ethical considerations, informed consent was obtained from all participants awaiting eCD. This consent process specifically addressed the ongoing CTG monitoring after the decision to perform an eCD to ensure that patients were fully aware of the procedures and of their right to decline unnecessary interventions. We ensured that all participants were provided with comprehensive information regarding the monitoring process, its purpose, and its potential implications, allowing them to make informed decisions about their care.

## Results

### Study participants

**Figure 4:**
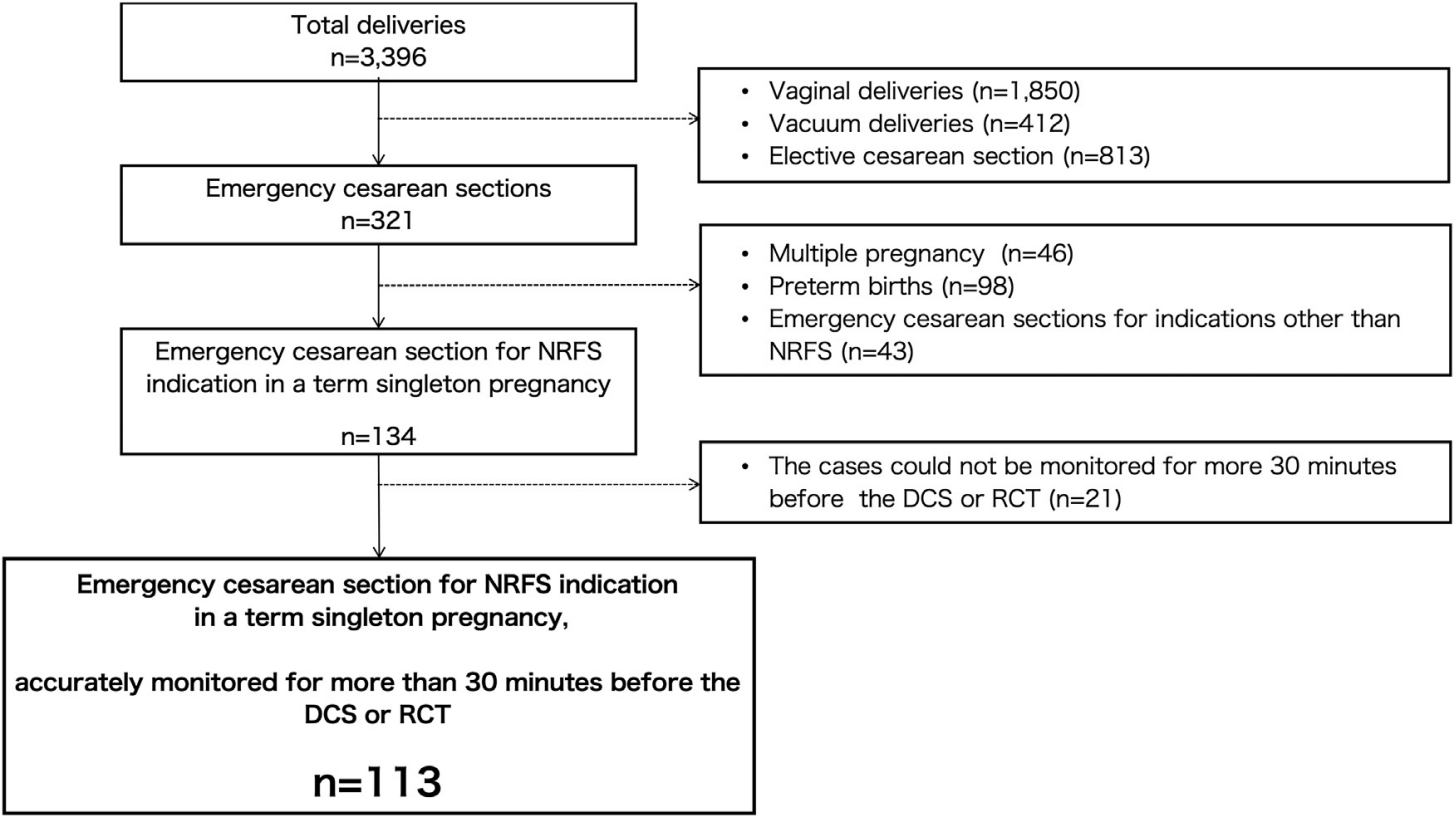
Flowchart of study participants *DCS*, decision of emergency cesarean delivery; *NRFS*, nonreassuring fetal status; *RCT*, removal of cardiotocography transducer.

There were 113 eligible patients (Figure 4). The patient characteristics are presented in Table 1. The results of the umbilical artery blood gas analysis were comparable among the level 3, 4, and 5 groups. Eleven (9.7%), 5 (4.4%), and 3 (2.7%) women had an umbilical cord artery blood pH of <7.2, <7.1, and <7.0, respectively, and 4 (3.5%) women were admitted to the neonatal intensive care unit (NICU) for neonatal asphyxia. These differences were not statistically significant among the level 3, 4, and 5 groups.

Ito. Integrated score index to predict fetal acidemia by intrapartum fetal heart rate monitoring is useful for predicting fetal acidemia in emergency cesarean delivery for nonreassuring fetal status during labor. Am J Obstet Gynecol Glob Rep 2024.

**Table 1:** Baseline characteristics of the study participants

Characteristic	All cases	Level 3	Level 4	Level 5	*P* value
Maternal age (y)	34.4±5.4	34.4±5.4	34.1±5.6	29.0±5.3	N.S.
Parity	0 (0–6)	0 (0–2)	0 (0–6)	0 (0–3)	N.S.
Gestational week	40+2 (37–42)	40+2 (37+0 to 42+0)	40+3 (37+0 to 42+0)	40+6 (40+3 to 41+6)	N.S.
Induction of labor, % (n/N)	67.3 (76/113)	71.1 (27/38)^a^	68.1 (49/72)^a^	0 (0/3)^b^	*P*<.05
Vacuum delivery, % (n/N)	8.0 (9/113)	5.3 (2/38)	9.7 (7/72)	0 (0/3)	N.S.
Time from decision of CD to delivery (min)	69 (20–242)	78 (33–242)^a^	65 (20–199)^b^	65 (52–135)^a,b^	*P*<.05
Time from removal of CTG transducer to delivery (min)	45 (15–99)	49 (21–76)^a^	37 (15–99)^b^	41 (34–56)^a,b^	*P*<.01
iPREFACE-DCS score	30 (4–88)	24 (5–45)^a^	31 (9–88)^b^	25 (14–33)^a,b^	*P*<.05
iPREFACE-RCT score	22 (0–90)	13 (0–64)^a^	24.5 (0–90)^b^	28 (0–40)^a,b^	N.S.
Birth weight (g)	2994±453	2899±476	3049±450	3066±112	N.S.
Fetal sex (male), % (n/N)	58.4 (66/113)	50 (19/38)	63.9 (46/72)	33.3 (1/3)	N.S.
Apgar score					
1 min	8 (2–9)	8 (2–9)	8 (2–9)	8 (8–8)	N.S.
5 min	9 (5–9)	9 (5–9)	9 (6–9)	9 (9–9)	N.S.
Chorioamnionitis, % (n/N)	23.0 (26/113)	18.4% (7/38)	23.6% (17/72)	66.7 (2/3)	N.S.
Meconium-stained amniotic fluid, % (n/N)	35.4 (40/113)	28.9 (11/38)	37.5 (27/72)	66.7 (2/3)	N.S.
Umbilical cord coiling, % (n/N)	39.8 (45/113)	47.4 (18/38)	34.7 (25/72)	33.3 (1/3)	N.S.
Umbilical artery blood acid-base analysis					
pH	7.29±0.1	7.30±0.1	7.29±0.1	7.31±0.0	N.S.
PCO_2_ (mm Hg)	49.4±11.7	48.8±14.9	49.9±9.9	44.7±7.3	N.S.
PO_2_ (mm Hg)	16.5±5.9	15.7±6.4	16.9±5.4	12.3±4.0	N.S.
HCO_3_^−^ (mmol/L)	23.6±3.9	23.4±5.5	23.8±2.7	22.1±3.5	N.S.
BE (mEq/L)	−3.0±3.8	−2.4±4.3	−3.2±3.6	−4.3±3.7	N.S.
Lactate (mmol/L)	3.1±2.2	2.7±2.4	3.2±2.2	3.7±2.3	N.S.
Umbilical artery blood pH<7.2, % (n/N)	9.7 (11/113)	2.6 (1/38)	12.5 (9/72)	0 (0/3)	N.S.
Umbilical artery blood pH<7.1, % (n/N)	4.4 (5/113)	2.6 (1/38)	5.6 (4/72)	0 (0/3)	N.S.
Umbilical artery blood pH<7.0, % (n/N)	2.7 (3/113)	2.6 (1/38)	2.8 (2/72)	0 (0/3)	N.S.
Admission to the NICU for neonatal asphyxia, % (n/N)	3.5 (4/113)	2.6 (1/38)	4.2 (3/72)	0 (0/3)	N.S.

Unless otherwise indicated, the data are presented as the mean (range) or as the mean ± standard deviation.

The superscript letters next to the right parenthesis indicate statistically significant differences between groups, denoted by different letters.

*BE*, base excess; *CD*, cesarean delivery; *CTG*, cardiotocography; *DCS*, decision of emergency cesarean delivery; *NICU*, neonatal intensive care unit; *N.S.*, not significant; *PCO_2_*, partial pressure of CO_2_; *PO_2_*; partial pressure of O_2_.

### Clinical use of the iPREFACE-RCT/DCS to predict an umbilical artery blood pH <7.2

**Figure 5:**
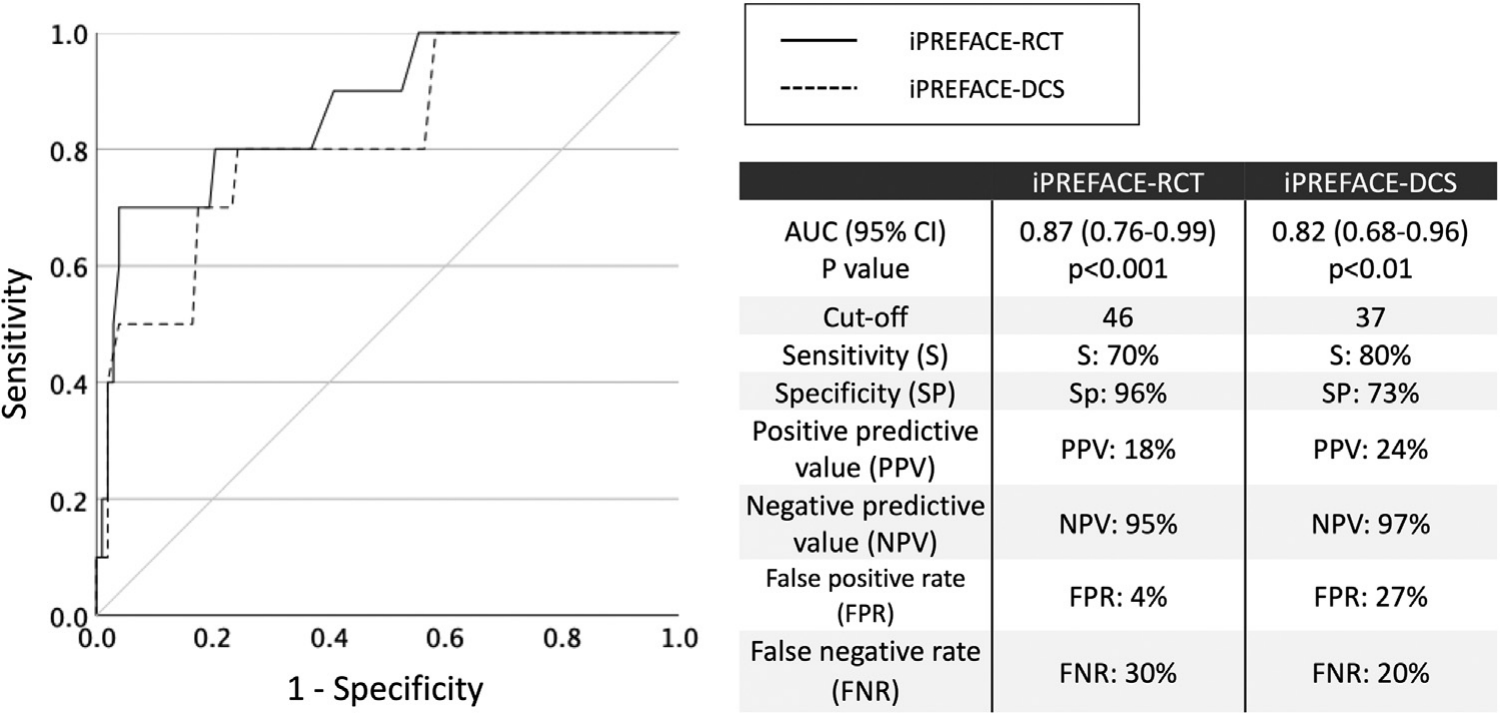
The predictive ability of the iPREFACE score for fetal acidemia. *AUC*, area under the receiver operating characteristic curve; *DCS*, decision of emergency cesarean delivery; *iPREFACE*, integrated score index to predict fetal acidemia by intrapartum fetal heart rate monitoring; *RCT*, removal of cardiotocography transducer.

The ROC-AUC value (95% confidence interval [CI]; *P* value) and cutoff value (specificity, sensitivity, positive predictive value, negative predictive value, false positive rate, and false negative rate) of the iPREFACE score as a predictive measure for an umbilical artery blood pH <7.2 were as follows: for the iPREFACE-RCT score, the ROC-AUC was 0.87 (95% CI, 0.76–0.99; *P*<.001) and the cutoff value was 46 points (70%, 96%, 18%, 95%, 4%, and 30%, respectively); for the iPREFACE-DCS score, the ROC-AUC was 0.82 (95% CI, 0.68–0.96; *P*<.01) and the cutoff value was 37 points (80%, 73%, 24%, 97%, 27%, and 20%, respectively) (Figure 5).

Ito. Integrated score index to predict fetal acidemia by intrapartum fetal heart rate monitoring is useful for predicting fetal acidemia in emergency cesarean delivery for nonreassuring fetal status during labor. Am J Obstet Gynecol Glob Rep 2024.

### Comparison of the two groups, stratified by the iPREFACE-RCT score of 46 points

A comparison of the iPREFACE-RCT group with ≥46 points and the iPREFACE-RCT groups with <46 points is presented in Table 2. No significant differences in maternal age, number of births, weeks of gestation, or birth weight were noted between the 2 groups. The number of vacuum deliveries was significantly higher in the iPREFACE-RCT ≥46 points group (*P*<.05). The time from surgical decision to birth and time from removal of the CTG transducer to birth were significantly shorter in the iPREFACE-RCT ≥46 points group (both *P*<.01). Umbilical artery blood gas analysis indicated that, in the iPREFACE-RCT ≥46 points group, values significantly trended toward mixed acidemia when compared with the values in the iPREFACE-RCT <46 points group. The number of cases with umbilical artery blood pH of <7.2, <7.1, and <7.0 and the number of NICU admissions for neonatal asphyxia were significantly higher in the iPREFACE-RCT ≥46 points group (*P*<.001, *P*<.01, *P*<.05, and *P*<.05, respectively).

**Table 2:** Characteristics of the two groups, stratified by the iPREFACE-RCT score of 46 points, in level 3 and 4 cases

Characteristic	iPREFACE-RCT score <46	iPREFACE-RCT score ≥46	*P* value
Maternal age (y)	33.8±5.6	36.2±4.6	N.S.
Parity	0 (0–6)	0 (0–2)	N.S.
Gestational week	40+2 (37+0 to 42+0)	40+3 (37+4 to 41+3)	N.S.
Induction of labor, % (n/N)	66.7 (68/102)	72.7 (8/11)	N.S.
Vacuum delivery, % (n/N)	5.9 (6/102)	27.3 (3/11)	*P*<.05
Time from decision of CD to delivery (min)	78 (22–242)	30 (20–69)	*P*<.01
Time from removal of CTG transducer to delivery (min)	47 (18–99)	23 (15–52)	*P*<.01
Birth weight (g)	3012±464	2885±374	N.S.
Fetal sex (male), % (n/N)	56.9 (58/102)	72.7 (8/11)	N.S.
Apgar score			
1 min	8 (2–9)	7 (2–8)	*P*<.05
5 min	9 (8–9)	9 (5–9)	N.S.
Chorioamnionitis, % (n/N)	23.5 (24/102)	18.2 (2/11)	N.S.
Meconium-stained amniotic fluid, % (n/N)	36.3 (37/102)	27.3 (3/11)	N.S.
Umbilical cord coiling, % (n/N)	40.2 (41/102)	27.3 (3/11)	N.S.
Umbilical artery blood acid-base analysis			
pH	7.32±0.04	7.13±0.16	*P*<.001
PCO_2_ (mm Hg)	47.8±8.30	64.7±21.5	*P*<.01
PO_2_ (mm Hg)	16.4±6.0	15.9±3.8	N.S.
HCO_3_^−^ (mmol/L)	24.0±3.8	20.8±43.8	*P*<.01
BE (mEq/L)	−2.1±2.4	−9.7±6.2	*P*<.001
Lactate (mmol/L)	2.4±1.2	7.2±4.6	*P*<.001
Umbilical artery blood pH<7.2, % (n/N)	2.9 (3/102)	63.6 (7/11)	*P*<.001
Umbilical artery blood pH<7.1, % (n/N)	2.0 (2/102)	27.3 (3/11)	*P*<.01
Umbilical artery blood pH<7.0, % (n/N)	1.0 (1/102)	18.2 (2/11)	*P*<.05
Admission to the NICU due to neonatal asphyxia, % (n/N)	2.0 (2/102)	18.2 (2/11)	*P*<.05

Unless otherwise indicated, the data are presented as the mean (range) or as the mean ± standard deviation.

*BE*, base excess; *CD*, cesarean delivery; *CTG*, cardiotocography; *DCS*, decision of emergency cesarean delivery; *NICU*, neonatal intensive care unit; *N.S.*, not significant; *PCO_2_*, partial pressure of CO_2_; *PO_2_*; partial pressure of O_2_.

**Figure 6:**
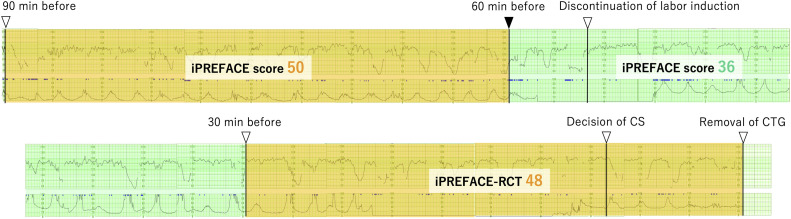
CTG findings in a participant with the most severe fetal acidemia. *CS*, cesarean delivery; *CTG*, cardiotocography; *iPREFACE*, integrated score index to predict fetal acidemia by intrapartum fetal heart rate monitoring; *RCT*, removal of cardiotocography transducer.

One patient's CTG, recorded 90 minutes before CTG transducer removal, and the case with the lowest umbilical cord artery blood pH in this study, is shown in Figure 6. An eCD was performed because of the NRFS indicator of repeated fetal heart rate waveforms at level 3 at 40 weeks and 5 days of gestation. The infant had a birth weight of 3207 g and an Apgar score of 2 and 5. The umbilical artery blood gas analysis readings were as follows: pH, 6.81; partial pressure of CO_2_, 104 mm Hg; partial pressure of O_2_, 10.9 mm Hg; bicarbonate level, 16.7 mEq/L; base excess, −20.3 mEq/L; and lactate level, 16.0 mmol/L. The infant was admitted to the NICU with the indication of asphyxia.

Ito. Integrated score index to predict fetal acidemia by intrapartum fetal heart rate monitoring is useful for predicting fetal acidemia in emergency cesarean delivery for nonreassuring fetal status during labor. Am J Obstet Gynecol Glob Rep 2024.

## Comments

### Principal findings

This study demonstrated the predictive ability of the iPREFACE score to detect fetal acidemia in cases of eCD for NRFS during labor. These findings could provide reference values and response guidelines in the context of the clinical applications of the iPREFACE score.

### Results in the context of what is known

The results of this study underscore the persistent challenge in predicting fetal acidemia as observed in previous studies. In contrast with the prevalent approach in most guidelines, which relies on a single abnormal waveform to determine delivery policy, this study suggests that the cumulative occurrence of abnormal waveforms indicates fetal distress. By quantifying the accumulation of fetal distress, the study aimed to enhance the predictive capacity of fetal acidemia, offering a potential indicator for making decisions regarding medical intervention during labor. The findings suggest that the accumulation of fetal distress could serve as a valuable indicator for guiding future medical interventions during labor.

### Clinical implications

Compared with the iPREFACE-RCT <46 points group, the iPREFACE-RCT ≥46 points group had more cases of umbilical cord artery blood pH of <7.1, umbilical cord artery blood pH of <7.0, and umbilical cord artery blood pH of <7.2 and more cases of NICU admission for neonatal asphyxia. These differences suggest that the iPREFACE score is a predictor of fetal acidemia. Furthermore, umbilical artery blood gas analysis findings were more likely to indicate metabolic acidosis in the iPREFACE-RCT ≥46 points group than in the iPREFACE-RCT <46 points group, which suggested that repeated decelerations caused hypoxemia and respiratory acidosis in the fetus, and further repeated decelerations gradually lead to metabolic acidosis.

### Research implications

The results of this study demonstrate that recurrent abnormal waveforms contribute to the accumulation of fetal distress, leading to a gradual transition of fetal arterial blood from respiratory acidosis to metabolic acidosis. The iPREFACE score has demonstrated high accuracy in detecting this transition and capturing the change, thereby showing the potential thereof to complement various CTG guidelines. To facilitate the clinical application of the iPREFACE score, it is essential to conduct retrospective studies involving a large number of cases of fetal acidemia and prospective studies to establish cutoff values for medical intervention during labor.

### Strengths and limitations

One limitation of this study is its retrospective nature with cases identified using the JSOG's 5-tier classification system. As shown in Figure 6, although 1 patient was initially diagnosed as level 3 during delivery, the attending obstetrician considered factors such as the speed and progress of delivery. Some time consequently elapsed before the final decision was made to proceed with rapid delivery. In instances when level 3 or 4 repeats over an extended period, fetal distress may accumulate because of repeated decelerations, potentially leading severe acidemia. In Figure 6, the iPREFACE score was already 50 points at 60 minutes before CTG transducer removal. Considering only the data within this CTG timeframe, an eCD could have at least been performed at this time. The iPREFACE score may be used to potentially prevent severe acidemia in such cases; therefore, further studies are required to determine clinically meaningful cutoff values, which should be validated in prospective studies.

### Conclusions

The iPREFACE score may aid clinicians in predicting an umbilical cord artery blood pH of <7.2 among women who undergo CD because of NRFS indicators. The iPREFACE score has the potential to support decision-making when level 3 or 4 persists in labor management based on the JSOGss 5-tier classification system.

## CRediT authorship contribution statement

**Ayumu Ito:** Writing – original draft, Project administration, Methodology, Investigation, Funding acquisition, Formal analysis, Data curation, Conceptualization. **Eijiro Hayata:** Writing – review & editing. **Hikari Kotaki:** Writing – review & editing. **Makiko Shimabukuro:** Writing – review & editing. **Mayumi Takano:** Writing – review & editing. **Sumito Nagasaki:** Writing – review & editing. **Masahiko Nakata:** Writing – review & editing, Supervision.
